# tRNA-derived fragment TRF365 regulates the metabolism of anterior cruciate ligament cells by targeting IKBKB

**DOI:** 10.1038/s41420-021-00806-4

**Published:** 2022-01-10

**Authors:** Dianbo Long, Yiyang Xu, Guping Mao, Ruobing Xin, Zengfa Deng, Hongyi Liao, Zhiwen Li, Zhi Yang, Baoxi Yu, Zhijian Yang, Aishan He, Ziji Zhang, Yan Kang

**Affiliations:** 1 Department of Joint Surgery, the First Affiliated Hospital of Sun Yat-sen University, Guangzhou, Guangdong 510080 China; 2 Guangdong Provincial Key Laboratory of Orthopedics and Traumatology, the First Affiliated Hospital of Sun Yat-sen University, Guangzhou, Guangdong 510080 China; 3 Department of Orthopedics, Shengli Clinical Medical College, Fujian Provincial Hospital, Fujian Medical University, Fuzhou, Fujian 350000 China

**Keywords:** Mechanisms of disease, Diseases

## Abstract

tRNA-derived fragments (tRFs) are new noncoding RNAs, and recent studies have shown that tRNAs and tRFs have important functions in cell metabolism via posttranscriptional regulation of gene expression. However, whether tRFs regulate cellular metabolism of the anterior cruciate ligament (ACL) remains elusive. The aim of this study was to investigate the role and action mechanism of tRFs in ACL cell metabolism. A tRF array was used to determine tRF expression profiles in different human ACL cells, and quantitative real-time polymerase chain reaction and fluorescence in situ hybridisation were used to determine *TRF365* expression. ACL cells were transfected with a TRF365 mimic or a TRF365 inhibitor to determine whether TRF365 regulates IKBKB expression. A rescue experiment and dual-luciferase reporter assay were conducted to determine whether the 3′-untranslated region (UTR) of *IKBKB* has a TRF365-binding site. TRF365 was weakly expressed in osteoarthritis (OA) ACL and interleukin-1β-treated ACL cells. IKBKB was highly expressed in OA ACL and interleukin-1β-treated ACL cells; transfection with the TRF365 mimic suppressed IKBKB expression, whereas transfection with the TRF365 inhibitor had the opposite effect. A dual-luciferase reporter assay showed that TRF365 silenced the expression of *IKBKB* by binding to its 3′-UTR. Thus, TRF365 regulates the metabolism of ACL cells by targeting IKBKB. In summary, TRF365 may provide a new direction for the study of ACL degeneration and on the pathophysiological process of OA.

## Introduction

Osteoarthritis (OA) is the most common degenerative joint disease, and its incidence has increased in recent years [1]. The occurrence of OA is closely related to several factors, but its pathogenesis remains unclear. Recent studies have found that inflammation and degeneration of all joint components promote the progression of OA, and ligament entheses may contribute to joint inflammation in OA [2]. The anterior cruciate ligament (ACL) is an intra-articular ligament that maintains the stability of the knee joint and plays an important role in the occurrence and development of OA. Recent studies have shown that there is an interrelation between ACL inflammation and degeneration, cartilage degradation, and dysbalanced subchondral bone remodelling [3–5].

Noncoding RNAs (ncRNAs) have been a focus of research in the last decade and have been extensively studied in OA progression [6]; however, the role of ncRNAs in ACL cell metabolism has not been sufficiently investigated. ncRNAs have been considered the most important mediators of gene expression at the posttranscriptional and epigenetic levels. Previous studies have shown that miRNAs, P-element-induced wimpy testis (PIWI)-interacting RNAs (piRNAs), and long ncRNAs are the most common regulatory ncRNAs [7]. However, transfer RNAs (tRNAs) and tRNA-derived small RNAs have been found to play important roles in the posttranscriptional control of gene expression [8]. tRNAs and tRNA-derived fragments (tRFs) are the most abundant small ncRNAs [9]. During early codon recognition, tRNAs deliver amino acids to the ribosome translation machinery to translate genetic information. Recently, a new function of tRNAs has been discovered; that is, they can give rise to tRFs, which have their own functions [8–10].

**Fig. 1:**
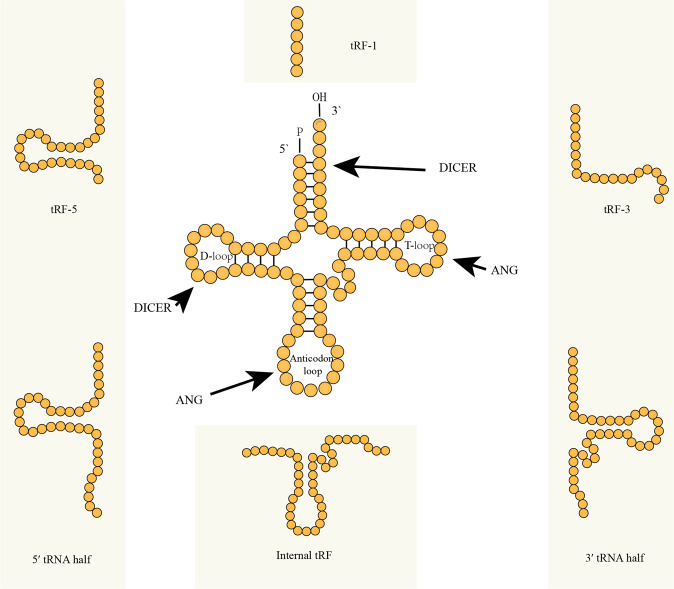
Transfer RNA (tRNA)structure and types of derived fragments. tRNA-derived fragments (tRFs) result from tRNA processing by Dicer and angiogenin (ANG) under stress conditions. Different types of tRFs are produced from tRNAs depending on the tRF start and end positions.

tRFs are processed by Dicer and angiogenin (ANG) under stress conditions, and their biogenesis is highly conserved and structure dependent, which suggests that tRFs may regulate metabolic functions within cells [11–13]. Depending on their start and end positions, tRFs can be classified into the following six major groups: tRF-1, tRF-3, tRF-5, internal tRF, 3′ tRNA half, and 5′ tRNA half (Fig. 1). tRFs mainly perform the following functions: regulate protein translation [14–16], silence genes via base pairing with target mRNAs [17, 18], sequester RNA-binding proteins [19, 20], and regulate transposable elements and ncRNA activity [21, 22]. For example, tRF-17-79MP9PP attenuates breast cancer cell invasion and migration via the THBS1/TGF-β1/SMAD3 axis [23]. tRF^GlnCTG^, which is induced by arterial injury, promotes vascular smooth muscle cell proliferation by negatively regulating the expression of the FAS cell surface death receptor [24]. A specific tRNA half (tiRNA), 5′ tiRNA-His-GTG, responds to hypoxia via the HIF1α/ANG axis and promotes colorectal cancer progression by regulating the LATS proteins [25].

The inhibitor of nuclear factor kappa B kinase subunit beta (IKBKB) is a key molecule in the NF-κB signalling pathway and functions by phosphorylating IκB, the inhibitor of the NF-κB transcription factor [26, 27]. Indeed, several signalling pathways that activate NF-κB converge at IKBKB [28]. Therefore, the IKBKB/NF-κB signalling pathway plays an important role in pathological processes of various diseases [29]. However, the underlying mechanisms of TRF365 and IKBKB in ACL cell metabolism have not been elucidated. In this study, we aimed to determine whether tRFs regulate ACL cell metabolism.

## Results

### Differential expression of tRFs among NA, normal, and IL-1β ACL cells

**Fig. 2:**
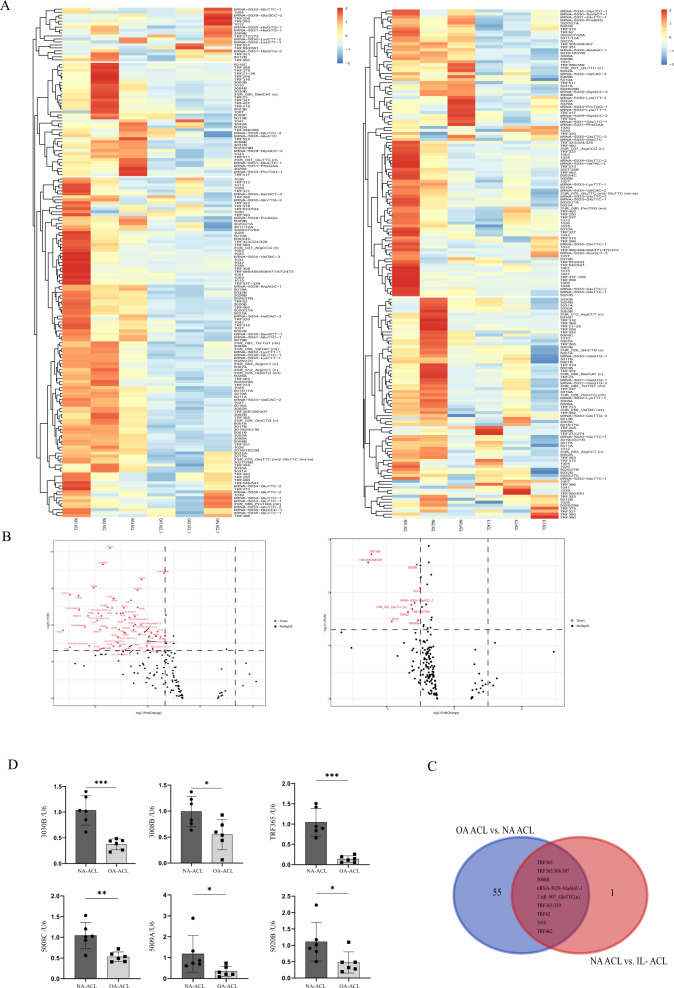
Expression of tiRNAs and tRFs in NA, normal, and IL-1β ACL cells. **A** Heatmap of differentially expressed tRFs among NA, osteoarthritis (OA), and IL-1β ACL cells. **B** Volcano plot of differentially expressed tRFs. Red dots represent significantly differentially expressed tRFs. **C** Nine tRFs with lower expression in both OA and IL-1β ACL cells than in NA ACL cells. **D** Expression of mature tRF-3008B, tRF-3030B, TRF365, tRF-5008C, tRF-5009A, and tRF-5020B in NA and OA ACL cells. **A**–**C** Each dot represents a value from a single experiment for one donor. The bar indicates the mean and 95% confidence interval of the values from five different donors per group. *P < 0.05, **P < 0.01, ***P < 0.001.

The tRF expression profiles in NA, OA, and IL-1β-treated ACL cells were analysed using a tRF array to identify the differentially expressed tRFs between paired samples within each of the three groups. The differentially expressed tRFs from all three paired samples are shown in Fig. 2A. Among the tRFs that were consistently differentially expressed in all three paired samples, 29 were down-regulated in OA, and IL-1β-treated ACL cells (Fig. 2B). We combined the two groups, and the results of the overlapped analysis of statistically significant differences in the expression of tRFs are shown in the Venn diagram in Fig. 2C. Finally, six tRFs were selected, and their expression was verified by quantitative real-time polymerase chain reaction (qRT-PCR), which showed low expression of tRF-3008B, tRF-3030B, TRF365, tRF-5008C, tRF-5009A, and tRF-5020B in OA and IL-1β ACL cells (Fig. 2D). These results were consistent with those of the tRF array analysis. Subsequently, TRF365 was selected for the subsequent analysis.

### Expression of TRF365 in OA and normal ACL cells

**Fig. 3:**
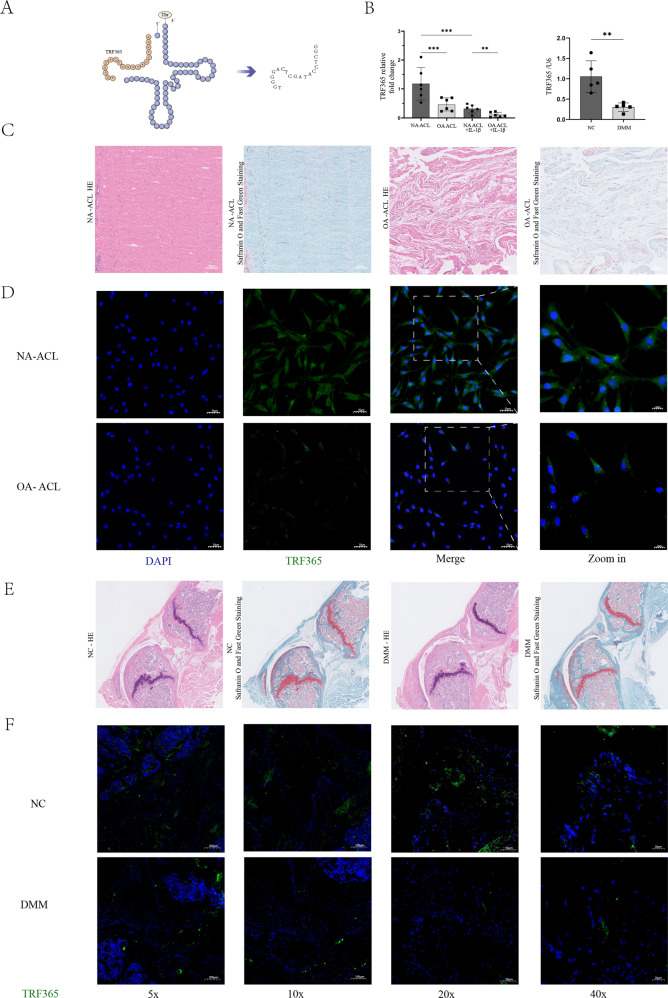
Expression of TRF365 in OA and NA ACL cells. **A** Structure and sequence of TRF365, derived from the internal region of tRNA^ThrTGT^. **B** Expression of TRF365 in OA and NA ACL cells, with and without IL-1β stimulation; expression of TRF365 in destabilisation of the medial meniscus (DMM) OA and normal control (NC) mouse ACL cells. **C** Hematoxylin and eosin (HE), and Safranin O and fast green staining showed histological differences between NA ACL and OA ACL. **D** RNA fluorescence in situ hybridisation analysis of TRF365 in OA and NA ACL cells. **E** HE and Safranin O and fast green staining showed DMM mice were successfully modelled. **F** RNA fluorescence in situ hybridisation analysis of TRF365 in DMM and NC mice. *P < 0.05, **P < 0.01, ***P < 0.001.

The tRF array analysis results showed that the expression of TRF365 was considerably down-regulated in OA ACL cells. The TRF365 sequence was derived from the 5′-end of tRNA^ThrTGT^ (Fig. 3A). qRT-PCR and immunofluorescence showed that the expression of TRF365 was considerably reduced in OA ACL cells compared with that in normal ACL cells (Fig. 3B, D). HE and Safranin O and Fast Green Staining also showed histological differences between NA ACL and OA ACL. Similar results were observed in ACL tissues from DMM and NC mice (Fig. 3E, F).

### Prediction of the target gene of TRF365 in ACL cells

**Fig. 4:**
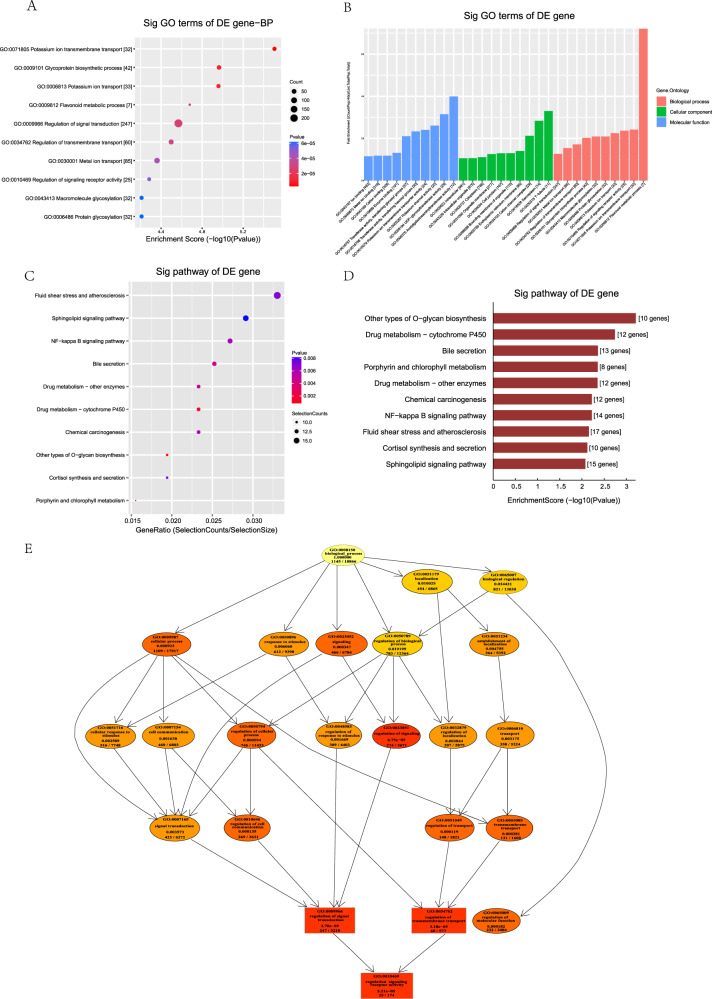
Bioinformatic analysis of TRF365. **A** Dot plot showing Gene Ontology (GO) enrichment for biological processes. **B** Bar plot showing significant GO terms as enrichment score values of significantly enriched pathways, including biological processes, cellular components, and molecular functions. **C** Dot plot showing gene ratios in the pathways. The size of the dot represents the number of genes in the significant target gene list associated with the biological process term, and the colour of the dot represents the *P* value. **D** Bar plot showing the top 10 enrichment score values of significantly enriched pathways. **E** GO-directed acyclic graph explanation. When a gene is annotated to a specific node, it is also considered annotated to the parent nodes. Top 10 terms with the lowest *P* values and their parents are shown in the GO-directed acyclic graph, with the more intense red representing higher significance.

After predicting the possible targets of TRF365 using TargetScan and miRanda, functional enrichment and pathway prediction of the assumed target genes were performed using the GO and KEGG pathway analyses. The results showed the potential of TRF365 to regulate the NF-κB signalling pathway. A combined analysis showed that *IKBKB* is a potential target gene of TRF365 (Fig. 4A–E).

### Expression of IKBKB in OA and IL-1β ACL cells

**Fig. 5:**
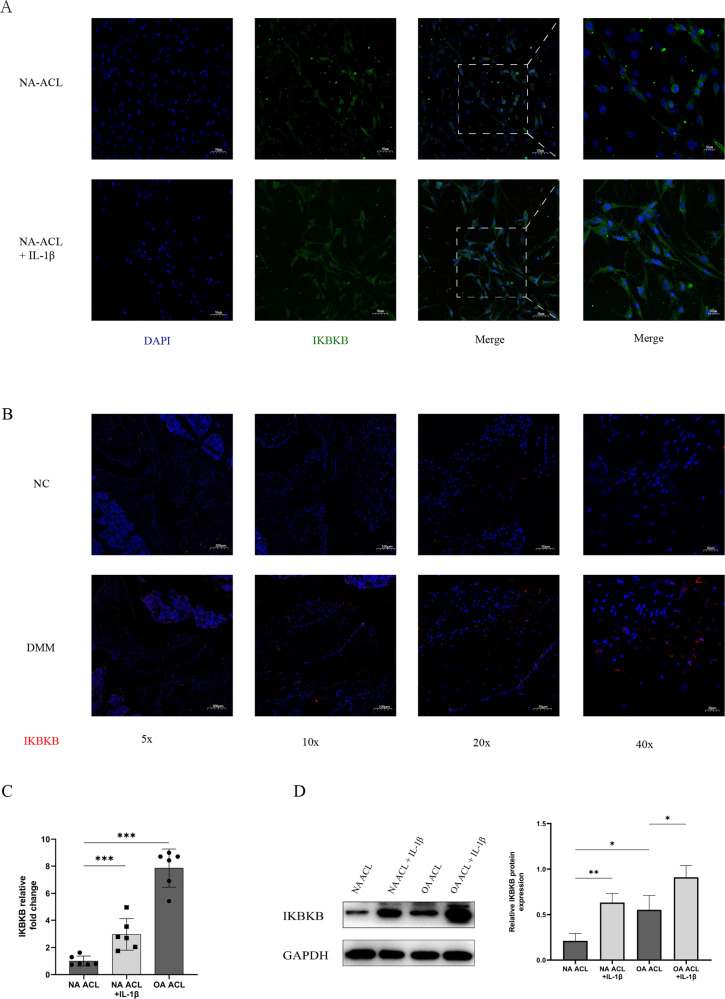
Expression of IKBKB in OA and NA ACL cells. **A**, **B** IKBKB expression detected by immunofluorescence. **C**, **D** Expression of IKBKB in OA and NA ACL cells, with and without IL-1β stimulation. *P < 0.05, **P < 0.01, ***P < 0.001.

To investigate the changes in IKBKB expression during OA progression, we examined IKBKB expression in normal, OA, and IL-1β ACL cells. IKBKB was highly expressed, at both mRNA and protein levels, in OA and IL-1β ACL cells compared with that in normal ACL cells (Fig. 5A, C, D). These results suggest that TRF365 expression was inversely correlated with that of IKBKB in human ACL cells. IKBKB was also highly expressed in ACL tissues from DMM mice than in those from NC mice (Fig. 5B).

### Regulation of IKBKB expression by TRF365 in ACL cells

**Fig. 6:**
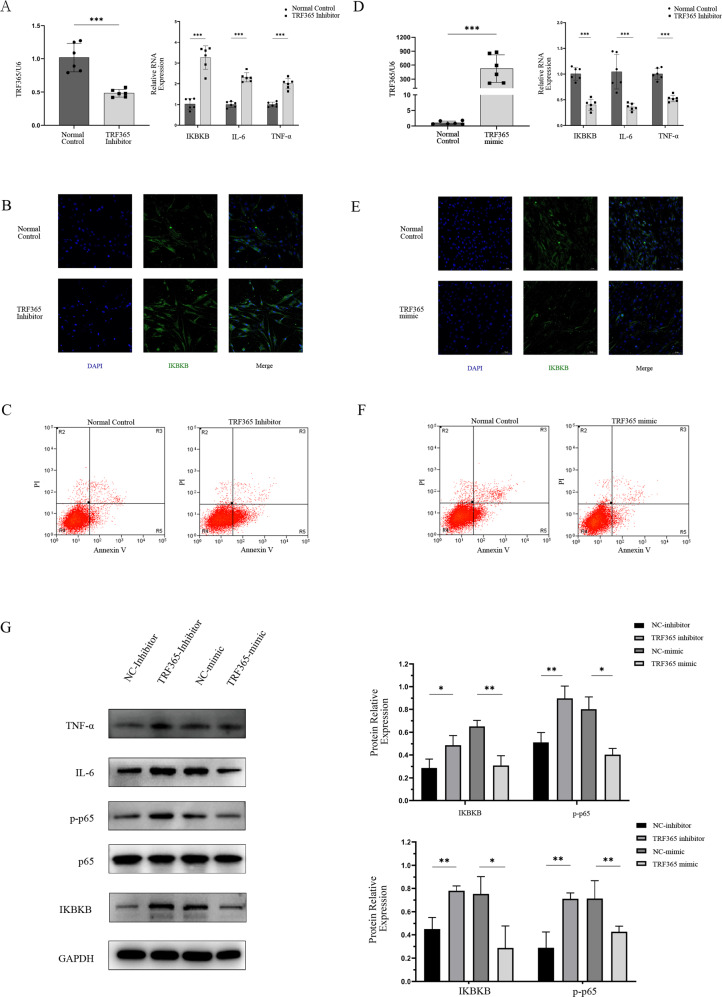
Regulation of IKBKB expression by TRF365 in ACL cells. **A**, **D** TRF365 expression detected by qRT-PCR after 24 and 48 h of ACL cell transfection with a TRF365 mimic or TRF365 inhibitor. **B**, **E** IKBKB and IL-6 expression detected by immunofluorescence. **G** IKBKB, IL-6, TNF-α, p-p65, p65 expression detected by western blotting. **C**, **F** Evaluation of the rate of cell apoptosis by flow cytometry after 48 h. *P < 0.05, **P < 0.01, ***P < 0.001.

To further investigate whether TRF365 regulates IKBKB expression in ACL cells, we transfected ACL cells with a TRF365 mimic and TRF365 inhibitor. Transfection with the TRF365 mimic increased and that with the TRF365 inhibitor decreased the expression of TRF365 (Fig. 6A, D). Moreover, TRF365 overexpression decreased the levels of *IKBKB*, *IL-6, TNFα*, whereas transfection with the TRF365 inhibitor considerably increased *IKBKB*, *IL-6, TNFα* expression (Fig. 6B, E). Similar trends in IKBKB, IL-6, TNFα, p-p65 expression were observed at the protein level (Fig. 6G). In addition, overexpression of TRF365 inhibited ACL cells apoptosis, whereas TRF365 knockdown had the opposite effect.

### Effects of *IKBKB* knockdown in ACL cells

**Fig. 7:**
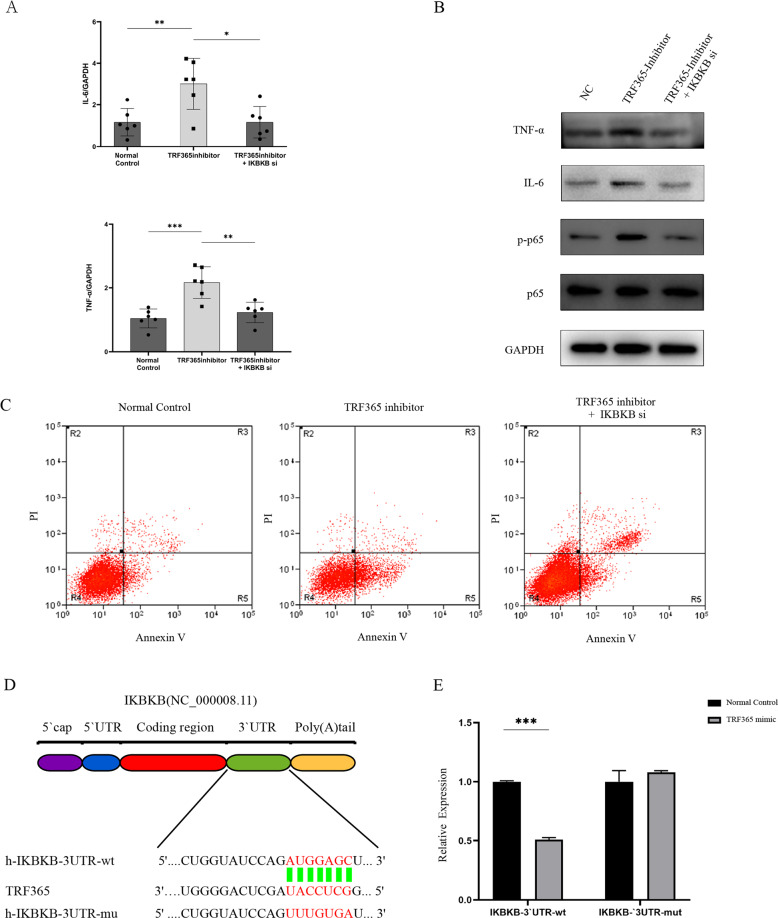
Effects of TRF365 binding to the 3′-UTR of *IKBKB* on translation. **A** qRT-PCR and (**B**) western blotting evaluation of the effects of co-transfection of an *IKBKB* siRNA and a TRF365 inhibitor. **C** Evaluation of the cell apoptosis rate by flow cytometry after 48 h. **D** Schematic illustration showing the flowchart of the prediction of the TRF365-binding site in *IKBKB* using a mutant. **E** Relative luciferase activity. *P < 0.05, **P < 0.01, ***P < 0.001.

To confirm that TRF365 regulates ACL cell metabolism by targeting *IKBKB*, the latter was knocked down using *IKBKB* small-interfering RNA (siRNA). The results showed that co-transfection of the *IKBKB* siRNA and the TRF365 inhibitor significantly decreased IL-6 expression, and the *IKBKB* siRNA blocked the TRF365 inhibitor-mediated up-regulation of *IKBKB* (Fig. 7A–C).

### Inhibition of luciferase reporter activity of the 3′-UTR of *IKBKB* mRNA by TRF365

To clarify the molecular mechanism underlying the regulation of IKBKB expression by TRF365, we analysed the sequence of the 3′-UTR of the human *IKBKB* mRNA. The results obtained using the predictive bioinformatics programmes miRanda and TargetScan showed that the 3′-UTR of human *IKBKB* contained a potential TRF365-binding site. Furthermore, a dual-luciferase reporter assay was performed to determine whether the 3′-UTR of *IKBKB* contains a TRF365 interaction sequence, and the sequence AUGGAGC was subsequently mutated into UUUGUGA (Fig. 7D). The luciferase activity of wild-type *IKBKB* was found to substantially vary when TRF365 was overexpressed. In contrast, the mutated binding sequence resulted in no change in the *IKBKB* 3′-UTR reporter activity when TRF365 was overexpressed (Fig. 7E). These results demonstrated that TRF365 reduced the luciferase activity by binding to the 3′-UTR of *IKBKB*.

## Discussion

ncRNAs have gained widespread interest and have been extensively studied in the last decade. The most important function of ncRNAs is the regulation of cell metabolism [6, 7]. tRFs are new small ncRNAs that have been discovered in the last few years. Recent studies have shown that tRNAs and tRFs also have important functions in cell metabolism. For example, tRF-5^Gln^, 19-nucleotide long, has been shown to inhibit protein translation in a sequence-independent manner [30]. tRFs can also silence genes by targeting mRNAs via base pairing. Thus, tRF-3017A regulates the tumour suppressor gene *NELL2* by forming an RNA-induced silencing complex with Argonaute proteins [31]. Furthermore, tRFs can interact with and sequester RNA-binding proteins from other RNAs. For example, tRF-5^Gln^ interacts with IGF2BP1 and sequesters it from *MYC*RNA, which leads to lowered stability of the latter [20]. tRFs also regulate transposable elements and ncRNA activity, as exemplified by the ability of long tRF-5s from mouse sperm and embryonic stem cells to globally repress MERVL-associated genes [32]. However, the importance of the modulation of tRF expression in human ACL cells remains unexplored.

In this study, we investigated whether a specific tRF, TRF365, regulates ACL cell metabolism. First, we analysed the expression of tRFs in different human ACL cells and selected TRF365 for further analyses; it is derived from the internal region at the 5′-end of mature tRNA^ThrTGT^. TRF365 showed low expression when ACL cells were under inflammatory conditions. To further explore the TRF365 function, its potential target genes were predicted. Notably, IKBKB can be activated by various kinases, such ad TAK1, NIK, MEKK, among others [27], which in turn phosphorylates IκB molecules, triggering their K48-linked polyubiquitination via βTrCP, with subsequent proteasomal degradation of IκB and release of active NF-κB. When NF-κB is activated, the expression of inflammatory factors such as IL-6 and TNF-α decreases. For example, miRNA-181 can down-regulate the NF-κB signalling pathway and reduce the expression of inflammatory factors TNF-α and IL-6 [33]. In contrast, miRNA-34a and miRNA-181a aggravate the NF-κB signalling pathway, as well as OA development [34].

A recent study has shown that miRNA-214-3p regulates the NF-κB signalling pathway by targeting *IKBKB*, and a decrease in miRNA-214-3p expression leads to an increase in *IKBKB* expression and activation of the NF-κB signalling pathway [28]. Another study has shown that platelet-rich plasma and *Sanguisorba officinalis* polysaccharide can increase the regenerative ability of ACL fibroblasts by blocking the TLR4/NF-κB pathway [35]. To clarify the expression pattern of *IKBKB* in the ACL, we compared *IKBKB* expression in normal and OA ACL cells and found that *IKBKB* was highly expressed in OA ACL cells but not in normal ACL cells. Furthermore, there was an inverse relationship between the expression levels of TRF365 and *IKBKB*, and a combined analysis showed that *IKBKB* is a potential target gene of TRF365. An increase in the cellular complement of TRF365 using a TRF365 mimic led to the down-regulation of IKBKB expression and a simultaneous decrease in the number of apoptotic ACL cells. Conversely, when TRF365 was suppressed by a TRF365 inhibitor, the expression of *IKBKB* and the number of apoptotic ACL cells increased. The rescue assay suggested that TRF365 regulated the NF-κB signalling pathway by targeting IKBKB. We subsequently determined that TRF365 silenced the expression of *IKBKB* by binding to its 3′-UTR. These results indicated that TRF365 post-transcriptionally regulated gene expression by targeting IKBKB in ACL cells. Recent studies have shown that tRF-3003a interacts with AGO complexes and performs miRNA-like functions [36], and tRF^GlnCTG^ regulates FAS in vascular smooth muscle cells via the same mechanism [24]. Therefore, we hypothesise that TRF365 silences genes by base pairing with *IKBKB* mRNA.

In conclusion, the results of this study indicate that the overexpression of TRF365 is essential for the suppression of IKBKB expression in human ACL cells. To the best of our knowledge, this is the first study to demonstrate the regulation of IKBKB by a tRF. These findings provide a new direction for the study of degeneration of ACL and pathophysiological process of OA and may yield additional tools for the clinical diagnosis and treatment of OA.

## Materials and methods

### Tissue collection and primary ACL cell isolation and culture

Normal ACL cells were obtained from two male and three female patients (age: 22 ± 4.53 years) with no history of OA or rheumatoid arthritis, who underwent ACL reconstruction because of ACL rupture. OA ACL cells were obtained from OA knee joints in two male and three female patients during total knee replacement surgery (age: 67.2 ± 9.09 years).

To isolate ACL cells, the ACL was cut into 1–3 mm pieces and washed three times with phosphate-buffered saline (Gibco Life Technologies, USA) containing 1% penicillin (100 IU/ml) and streptomycin (100 μg/ml) solution (Gibco Life Technologies). The tissue was treated with collagenase type I (1 mg in 1 ml of medium per gram of tissue), which was sterilised by passing through a 0.2 μm filter (C0130; Sigma–Aldrich, USA), at 37 °C for 8 h. Any nondigested tissue was removed using a 70-µm cell strainer (BS-70-XBS; Biosharp, China). The cells that were passed through the cell strainer were centrifuged at 1500 rpm for 3 min and then seeded in a T75 flask [37, 38].

ACL cells were cultured in Dulbecco’s modified Eagle’s medium/Ham’s F-12 nutrient mixture (Gibco Life Technologies) containing 10% foetal bovine serum (Gibco Life Technologies) and 1% penicillin/streptomycin solution. For IL-1β treatment, ACL cells were cultured in the same medium containing IL-1β (5 ng/ml) for 48 h. Cells were incubated at 37 °C in a humidified atmosphere of 5% CO_2_, and the medium was changed every 2 days.

Knee tissue section from destabilization of the medial meniscus (DMM) mice and normal control (NC) mice were kindly provided by Hongyi Li from the Department of Joint Surgery, the First Affiliated Hospital of Sun Yat-sen University, Guangzhou, Guangdong, China [39]. Male wild-type C57 BL/6 J (GemPharmatech, Co., Jiangsu, China) were housed under specific pathogen-free conditions and used in experiments at 10 weeks of age. The mice were subjected to DMM surgery of the right knees and their left knees were sham operated as control. After 8 weeks, the mice were sacrificed and the knee joints were harvested from these mice for further experiments.

### RNA extraction, reverse transcription, and qRT-PCR

The total RNA was extracted using the RNeasy mini kit (Qiagen, Germany) following the manufacturer’s instructions. The extracted RNA was quantified using a NanoDrop spectrophotometer (NanoDrop Technologies, USA), and cDNA was synthesised from the total RNA using the PrimeScript RT master mix (Takara, Japan). The rtStar tRF & tiRNA Pretreatment Kit (Cat. #AS-FS-005; Arraystar) was used for miRNA sample pretreatment. The rtStar First-Strand cDNA Synthesis Kit with 3′ and 5′ adaptors (Cat. #AS-FS-003; Arraystar) was used for cDNA synthesis. qRT-PCR was performed using the SYBR Green Pro Taq HS qPCR Kit II with ROXdye (Accurate Biology, China) according to the manufacturer’s instructions on an ABI real-time PCR system (ViiA 7Dx; Applied Biosystems, USA). The primers used are listed in Supplementary Materials Table S1. The mRNA transcript levels were normalised to those of the reference gene *GAPDH*, and U6 small nuclear RNA was used as an endogenous control for tRF quantification in cells. Relative gene expression levels were calculated using the 2^−ΔΔCt^ method. All experiments were performed at least three times.

### tRF array analysis

A tRF expression profiling assay was conducted using the nrStar Human tRF & tiRNA PCR array and nine samples: three normal ACL cell samples from three donors, three normal ACL cell samples treated with IL-1β (5 ng/ml) for 24 h, and three OA ACL cell samples from three donors. The array analysis and data evaluation were performed by KangChen Biotech (Shanghai, China). R4.0.0 software was used to identify the differentially expressed tRFs among the groups.

### Target gene prediction and bioinformatic analysis

To determine the potential target genes of TRF365, online analysis tools, miRanda (http://www.microrna.org/microrna/home.do) and TargetScan (http://www.targetscan.org/vert_72/), were used. The bioinformatic analyses were Gene Ontology function analysis (http://www.geneontology.org/) and Kyoto Encyclopaedia of Genes and Genomes (KEGG) pathway analysis to predict the pathways for TRF365.

### Transfection of the *IKBKB* siRNA and the TRF365 mimic and inhibitor

ACL cells were seeded in six-well plates and allowed to grow to 80% confluence. The cells were transfected with a *Homo sapiens* TRF365 mimic and inhibitor (RiboBio, China) and an *IKBKB* siRNA (RiboBio) using Lipofectamine 3000 (Invitrogen, USA) according to the manufacturer’s instructions. Nonspecific oligonucleotides were used as a tRF mimic negative control (NC), tRF inhibitor NC, and NC siRNA. The cells were harvested after 24 h of incubation for qRT-PCR and 48 h of incubation for western blotting.

### Immunofluorescence and RNA fluorescence in situ hybridisation

To detect TRF365 expression, sections and cells on coverslips were subjected to in situ hybridisation analysis. Briefly, fixed cells were permeabilised with 0.1% Triton X-100 and 10 mM VRC CSK buffer, and then treated with RNase R at 37 °C for 15 min and fixed again. The fixed cells were dehydrated using 70%, 80%, and 100% ethanol. A TRF365 probe was used for hybridisation, which was performed at 37 °C overnight. The slides were successively washed with 4×, 2×, and 1× SSC buffer and then stained with 4′,6-diamidino-2-phenylindole (2 mg/ml) for 10 min at 37 °C. Images were acquired using a confocal microscope (LSM780; Carl Zeiss, Germany). Fluorescence intensity was analysed using ImageJ software. The fluorescent oligonucleotide probe for TRF365 was synthesised by Servicebio (Wuhan, China), and its sequence was as follows: 5′-biotin label-GGCTCCATAGCTCAGGGG-3′ (two-tailed 6-carboxyfluorescein).

Immunofluorescence was performed using standard methods, and it included incubation with a primary antibody against IKBKB (1:200 dilution; Proteintech, China), followed by incubation with a secondary antibody and counterstaining with 4′,6-diamidino-2-phenylindole. Images were captured using a confocal laser microscope (Olympus, Japan) at different magnifications.

### Flow cytometry assay for the analysis of apoptosis

ACL cells were seeded in six-well plates and allowed to grow to 80% confluence. The cells were then transfected with the TRF365 mimic (50 nM) or TRF365 inhibitor (100 nM) and cultured in Dulbecco’s modified Eagle’s medium/nutrient mixture F12 containing 10% foetal bovine serum, 1% penicillin/streptomycin, and IL-1β (5 ng/ml) for 48 h. The cells were harvested, and then washed with cold phosphate-buffered saline, dissociated into single cells, and stained with propidium iodide and fluorescein isothiocyanate-conjugated Annexin V using the FITC Annexin V Apoptosis Detection Kit I (BD Biosciences, USA). Within 1 h of staining, the cells were examined by flow cytometry, and the data were analysed using FlowJo.

### Western blotting

Western blotting was performed as previously described [40]. Proteins were resolved on 10% sodium dodecyl sulphate-polyacrylamide gel and transferred on to polyvinylidene difluoride membranes (Millipore, Bedford, MA, USA). After blocking with 5% non-fat milk in Tris-buffered saline plus 0.1% Tween 20, the membranes were incubated with primary antibodies against IKBKB (1:1000; #2678, Cell Signalling Technology), IL-6 (1:1000; #66146, Proteintech) and TNF-α (1:1000; #17590) both from Proteintech, and phoshorylated-p65 (1:1000; #3033), p65 (1:1000; #8242), and GAPDH (1:2000; #2118) all from Cell Signalling Technology (USA), which was used as the internal control. After washing, the blots were incubated with the corresponding secondary antibodies (Cell Signalling Technology) at room temperature for 1 h and visualised using an ECL Chemiluminescence Kit (Millipore).

### Dual-luciferase reporter assay

To determine the target regulatory relationship between TRF365 and *IKBKB* mRNA, a dual-luciferase reporter assay was performed using SW1353 cells. The 3′-UTR of *IKBKB* mRNA with the TRF365-binding site or its mutant construct was inserted into the luciferase reporter plasmid. The luciferase reporter was co-transfected with the *IKBKB*3′-UTR fusion vector and the TRF365 mimic or the corresponding NC. The cells were harvested 48 h later. The dual-luciferase reporter assay (Promega, USA) and a Synergy H1 microplate reader (BioTek Instruments, USA) were used to detect firefly and *Renilla* luciferase activities.

### Statistical analysis

Continuous data are presented as mean ± standard deviation. The one-way analysis of variance or Student’s *t* test was used to determine significant differences among or between groups, respectively. Results with *P* < 0.05 were considered statistically significant. Data were analysed using SPSS statistical software version 23.0 (IBM Corp., USA). All experiments were performed at least three times.

## Supplementary information

STable 1

## Data Availability

The datasets for the current study are available from the corresponding author upon reasonable request.

## Availability of data and materials

All data generated or analysed during this study are included in this published article.
