# Supplementary material for: tRNA-derived fragment TRF365 regulates the metabolism of anterior cruciate ligament cells by targeting IKBKB
Source: Cell Death Discov. 2022 Jan 10;8:19. doi: 10.1038/s41420-021-00806-4 (PMC8748987; doi:10.1038/s41420-021-00806-4)
Supplement: Supplementary file 1 — STable 1 [file 41420_2021_806_MOESM1_ESM.docx]

**supplement Table S1**. Primers sequences for qRT-PCR.

|  | **Sequence** |
| --- | --- |
| 3008B | CGGGCGGAAACACCAAAAA |
| 3030B | ATTCCGGCTCGAAGGACCAA |
| 5008C | GTTGGTGGTATAGTGGTGAG |
| 5009A | GGGCTTCTGTAGTGTAGAAAA |
| 5020B | GTTCCATAGTGTAGTGGTTATC |
| TRF365 | GCTCCATAGCTCAGGGGTAA |
| IKBKB-Forward primer | GTCTTTGCACATCATTCGTGGG |
| IKBKB-Reversed primer | GTGCCGAAGCTCCAGTAGTC |
| IL-6-Forward primer | ACTCACCTCTTCAGAACGAATTG |
| IL-6-Reversed primer | CCATCTTTGGAAGGTTCAGGTTG |
| TNF-α-Forward primer | CCGGGCAACAATGTCCAAAAG |
| TNF-α-Reversed primer | AGGACGACTGTTCAGCACG |
| U6-Forward primer | GGAACGATACAGAGAAGATTAGC |
| U6-Reversed primer | TGGAACGCTTCACGAATTTGCG |
| GAPDH-Forward primer | AGGTCGGTGTGAACGGATTTG |
| GAPDH-Reversed primer | GGGGTCGTTGATGGCAACA |
